# Mcl-1 Is a Novel Target of miR-26b That Is Associated with the Apoptosis Induced by TRAIL in HCC Cells

**DOI:** 10.1155/2015/572738

**Published:** 2015-05-21

**Authors:** Chunlin Jiang, Jianting Long, Baoxian Liu, Xiaoyan Xie, Ming Kuang

**Affiliations:** ^1^Department of Hepatobiliary Surgery, Division of Interventional Ultrasound, The First Affiliated Hospital, SUN Yat-Sen University, Guangzhou 510080, China; ^2^Department of Medicinal Oncology, The First Affiliated Hospital, SUN Yat-Sen University, Guangzhou 510080, China; ^3^Department of Medical Ultrasonics, Institute of Diagnostic and Interventional Ultrasound, The First Affiliated Hospital, SUN Yat-Sen University, Guangzhou 510080, China

## Abstract

*Aim*. To investigate the role of miR-26b and Mcl-1 in TRAIL-inducing cell death in hepatocellular carcinoma. *Methods*. The expression of miR-26b and Mcl-1 in HCC was detected by RT-qPCR and western blot. The regulation of Mcl-1 by miR-26b was determined by luciferase reporter assay. MTT and flow cytometry were employed to detect the cell viability and apoptosis. *Results*. miR-26b is commonly downregulated in HCC cell lines compared with the LO2 cell line. In contrast, the Mcl-1 expression is upregulated in HCC cell lines. Bioinformatic analysis identified a putative target site in the Mcl-1 mRNA for miR-26b and luciferase reporter assay showed that miR-26b directly targeted the 3′-UTR (3′-Untranslated Regions) of Mcl-1 mRNA. Transfection of miR-26b mimics suppressed Mcl-1 expression in HCC cells and sensitized the cancer cells to TRAIL (tumor necrosis factor-related apoptosis-inducing ligand) cytotoxicity. In addition, transfection of HCC cells with Mcl-1 expression plasmid abolished the sensitization effect of miR-26b to TRAIL-inducing apoptosis. *Conclusions*. Our study showed that miR-26b was a negative regulator of Mcl-1 gene and sensitized TRAIL-inducing apoptosis in HCC cells, suggesting that the miR-26b-Mcl-1 pathway might be a novel target for the treatment of HCC.

## 1. Background

Hepatocellular carcinoma (HCC) is a worldwide problem hazard to human health, which has poor prognosis, short survival time, and is with the 3rd mortality rate within all cancers [1]. Researches in the past few decades revealed that a number of factors can increase the risk of developing HCC, such as hepatitis virus infection, metabolic diseases, and liver fibrosis [2, 3]. However, the pathogenesis of HCC is still not very clear, and high expression of some protective proteins in cells may be an important cause of HCC incidence [4].

Mcl-1 is an antiapoptotic member in the Bcl-2 family proteins, which contains three BH (Bcl-2 homology) domains and plays antiapoptotic effects by binding to proapoptotic proteins Noxa, puma, bim, bid, and so forth [5]. Researches revealed that overexpression of Mcl-1 could be against the apoptotic stimulus such as TRAIL- (tumor necrosis factor-related apoptosis-inducing ligand-) inducing cell death. In contrast, knockdown of Mcl-1 enhanced the TRAIL cytotoxicity [6]. Mcl-1 seems to be more widely distributed within the cell than other Bcl-2 proteins. Besides to mainly localize in the mitochondrial membrane, Mcl-1 has also been found in the nucleus and the cytoplasm [7]. Mcl-1 is highly expressed in a variety of human tumor tissues, such as breast cancer, colon cancer, lung cancer, ovarian cancer, prostate cancer, kidney cancer, and liver cancer [8, 9]. Although overexpression of Mcl-1 does not directly promote the proliferation of tumor cells, its ability to suppress apoptosis plays a key role for cancer cell to protect against the apoptosis-inducing effect caused by toxic factors. Protection from apoptosis via overexpression of Mcl-1 in tumor cells may represent a significant barrier to the effectiveness of chemotherapeutic agents [10, 11].

MicroRNAs (miRNAs) are a class of small, endogenous, noncoding, single-stranded RNAs of 19–25 nucleotides cleaved from 70–100 nucleotide hairpin pre-miRNA precursors. miRNAs bind to the complementary sequences of the target mRNA 3′-UTR and then induce the mRNA degradation or translational repression, regulating the expression of the target genes [12, 13]. Currently, there are over 1,000 miRNAs that have been identified in human cells, regulating up to 60% of protein-coding genes in human genome [14]. miRNAs regulate a variety of physiological and pathological cellular processes, including cell growth, differentiation, proliferation, and apoptosis [15]. Recent studies have shown that about half of the human miRNAs are located in cancer-associated genomic regions and frequently dysregulated in cancer, suggesting that miRNAs play a key role in oncogenesis [16]. It is reported that the expression of miR-26b is dysregulated in many kinds of cancers, which is profoundly involved in oncogenesis, epithelial-mesenchymal transition, and resistance to medical drugs [17, 18]. However, the function of miR-26b in HCC is still little known.

In the present study, bioinformatics analysis predicts that miR-26b potentially targets the Mcl-1 3′-UTR, which is confirmed by the luciferase assay. We demonstrated that miR-26b could downregulate Mcl-1 expression in HCC cells by directly targeting the 3′-UTR of Mcl-1 mRNA. We also found that transfection of miR-26b mimics augments the TRAIL-inducing apoptosis and then we explored some underlying molecular mechanisms.

## 2. Materials and Methods

### 2.1. Reagents and Antibodies

miR-26b mimics and negative control oligonucleotide (NC oligo) were purchased from RiboBio Company (China). The sequences of the above RNA oligos were as follows: miR-26b mimics: 5′-UUCAAGUAAUUCAGGAUAGGU-3′; NC oligo: 5′-UGUAAUAAUGGAACUCGGAUU-3′. 3-(4,5-Dimethylthiazol-2-yl)-2,5-diphenyltetrazolium bromide (MTT), trypan blue, and Annexin V-FITC Apoptosis Detection Kit were obtained from Sigma-Aldrich (USA). Antibodies for rabbit anti-human Mcl-1 and rabbit anti-human *β*-actin were purchased from Cell Signaling (USA). Human recombinant TRAIL was obtained from R&D Systems (USA).

### 2.2. Cell Culture

Human HCC cell lines (HepG2, Hep3B, PLC, and Huh7) and the human embryo liver cell line (L02) were from the Institute of Biochemistry and Cell Biology, Chinese Academy of Sciences (Shanghai, China), and cultured in DMEM basic medium (Gibco, USA) with 10% fetal bovine serum (FBS, Gibco, USA) at 37°C in a humidified 5% CO_2_ incubator.

### 2.3. Mcl-1 mRNA Expression Assay by Quantitative PCR (qPCR)

Total RNA was extracted with TRIzol (Invitrogen, USA) and reverse-transcribed into cDNA with M-MLV Reverse Transcriptase (Invitrogen, USA) following the manufacturer's instructions. qPCR was performed using SYBR Green (TaKaRa, Japan) on the Applied Biosystems 7500 Real Time PCR System, taking GAPDH mRNA as internal control and the relative level of Mcl-1 expression was determined with the 2^−ΔΔCT^ method [19]. Quantitative PCR primer sequences are as follows. Mcl-1 forward: 5′-CGACGGCGTAACAAACT-3′, Mcl-1 reverse: 5′-GGAAGAACTCCACAAACCC-3′; GAPDH forward: 5′-TGCCAAATATGATGACATCAAGAA-3, GAPDH reverse: 5′-GGAGTGGGTGTCGCTGTTG-3′.

### 2.4. miR-26b Expression Assay by Quantitative PCR (qPCR)

Total RNA was extracted with TRIzol. Since the miR-26b is only composed of 22 nucleotides, we used stem-loop RT-qPCR (reverse transcription-qPCR) method [20]. Taking U6 small nuclear RNA (snRNA U6) as internal control, the relative level of miR-26b expression was determined with the 2^−ΔΔCT^ method. The primer sequences are as follows. miR-26b RT primer: 5′-CTCAACTGGTGTCGTGGAGTCGG CAATTCAGTTGAGACCTATCC-3′; U6 snRNA RT primer: 5′-AACGCTTCACGAATTTGCGT-3′.

### 2.5. Plasmid Construction

Total RNA was isolated by TRIzol. RNA was then reverse-transcribed into cDNA with M-MLV Reverse Transcriptase using the primer of oligo(dT) (Takara, Japan). The 3′-UTR region of human Mcl-1 (NM_001197320) was amplified by PCR using the cDNA as a template and cloned into the pMIR-REPORT miRNA Expression Reporter Vector System (pMIR, Life Technologies, USA). The recombinant plasmid was named pMIR-Mcl-1. The mutant plasmid was created by mutating the seed regions of the miR-26b-binding sites (UACUUGA to UAGAAGA) by using site-directed mutagenesis kit (Takara, Japan) and named pMIR-Mcl-1-M. The open reading frame of Mcl-1 gene without 3′-UTR was amplified by PCR with the cDNA as template and cloned into the pEGFP-N1 vector (Clontech, USA) and the resulting plasmid was named pEGFP-Mcl-1.

### 2.6. Transient Transfection

HCC cells were seeded in 6-, 48-, or 96-well plates and cultured for 16 h; then the luciferase reporters (pMIR, pMIR-Mcl-1, pMIR-Mcl-1-M), eukaryotic expression vector (pEGFP-N1, pEGFP-Mcl-1), and RNA oligos (NC oligo or miR-26b mimics) were transiently transfected into the HCC cells with Lipofectamine 2000 reagent (Invitrogen, USA) following the manufacturer's instructions.

### 2.7. Luciferase Assay

HepG2 cells were seeded in 48-well plates for 16 h; then the Firefly luciferase reporters (pMIR, pMIR-Mcl-1, or pMIR-Mcl-1-M, 0.4 *μ*g/well) and Renilla luciferase pRL-TK vector (Promega, USA, 20 ng/well) as well as miR-26b (or NC oligo, 10 pmol/well) were transfected into the cells for 24 h. Then, the cells were lysed and the Firefly and Renilla luciferase activities were measured by Dual-Luciferase Reporter System (Promega, USA) according to the manufacturer's instructions. Firefly luciferase activity was normalized to the Renilla luciferase activity. Results were represented as the ratio between the various treatments and the NC control.

### 2.8. Western Blot

HCC cells as well as LO2 cells (normal hepatic cell line) were seeded in 6-well plates for 16 h; then the cells were transfected with RNA oligos (NC oligo or miR-26b mimics, 100 pmol/well) with/without plasmid (pEGFP-Mcl-1 or pEGFP-N1, 4.0 *μ*g/well) for 24 h. After treatment, the cells were collected and the total proteins were extracted using RIPA lysis buffer (Cell Signaling, USA). The protein concentrations were determined by BCA assay (Pierce, USA); then the proteins were separated on a 12.5% SDS-PAGE and transferred to the PVDF membranes (Millipore, USA). The membranes were then incubated with primary antibodies of rabbit anti-human Mcl-1 and rabbit anti-human *β*-actin. Then, after incubation with horseradish peroxidase-conjugated secondary antibody of goat anti-rabbit IgG, protein bands were visualized using enhanced chemiluminescence detection kit (Pierce, USA).

### 2.9. Cell Viability Assay

HepG2 cells were seeded into 96-well plates for 16 h and then transfected with the miR-26b or NC oligo (5 pmol/well) with/without plasmid (pEGFP-Mcl-1, pEGFP-N1, 0.2 *μ*g/well) for 24 h. TRAIL was then added (5 ng/mL final) in fresh media and the cells were treated for another 24 hours. Cell viability was measured by MTT assay [21]. Results were represented as the ratio between the various treatments and the NC control.

### 2.10. Measurement of Cell Death and Apoptosis

HepG2 cells were seeded into 6-well plates for 16 h and then transfected with the miR-26b or NC oligo (100 pmol/well) with/without plasmid (pEGFP-Mcl-1, pEGFP-N1, 4.0 *μ*g/well) for 24 h. TRAIL was then added (5 ng/mL final) in fresh media and the cells were treated for another 24 hours. Cell death was measured by trypan blue exclusion assay [22]; the cell death rate was determined by calculating staining cells to total cells under optical microscope. Cell apoptosis was measured using Annexin V-FITC Apoptosis Detection Kit according to the manufacturer's instructions and analyzed using flow cytometry.

### 2.11. Statistical Analysis

SPSS 13.0 software was used for statistical analysis. Data were presented as the mean ± SD and derived from at least three independent experiments. Comparisons between two groups were made by Student's *t*-test, and comparisons among three groups were made with ANOVA. *P* values less than 0.05 were considered to be of statistical significance.

## 3. Results

### 3.1. HCC Cells Express High Level of Mcl-1 and Low Level of miR-26b

**Figure 1:**
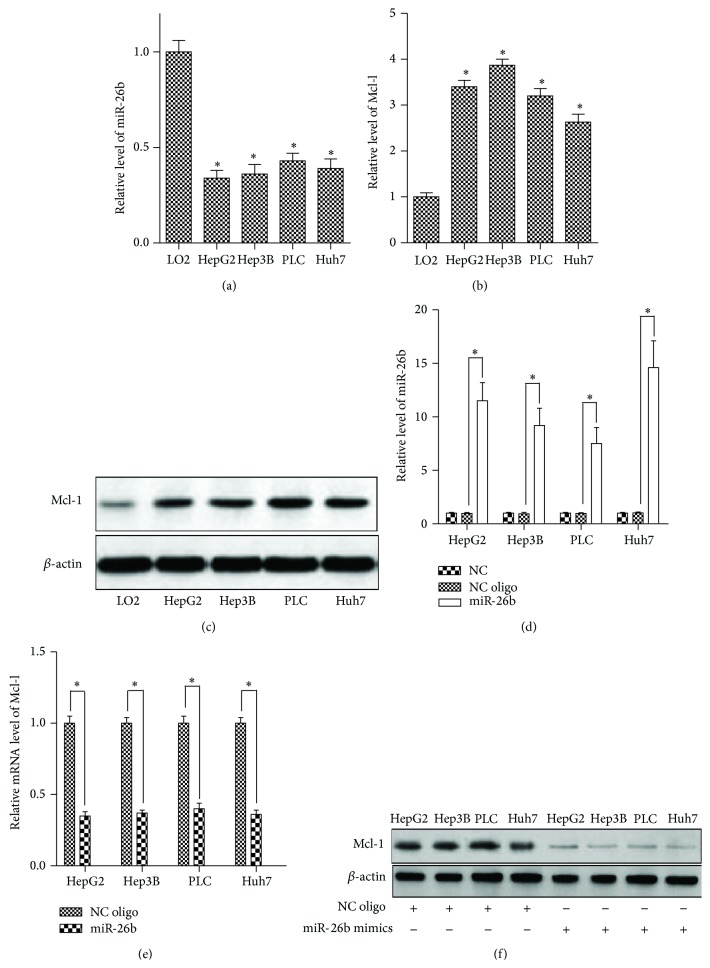
HCC cell lines express high level of Mcl-1 and low level of miR-26b. Three independent experiments were performed. (a) The miR-26b expression levels of L02, HepG2, Hep3B, PLC, and Huh7 cells were detected by qPCR. ^*∗*^ *P* < 0.05 versus LO2. (b) qPCR analysis for Mcl-1 mRNA expression level in all the above cell lines. ^*∗*^ *P* < 0.05 versus LO2. (c) Western blot analysis for Mcl-1 protein expression level in all the above cell lines. (d) qPCR analysis for miR-26b levels in HCC cells after transfection with miR-26b mimics or NC oligonucleotide for 6 h. (e) qPCR analysis for Mcl-1 mRNA expression in HCC cells after transfection with miR-26b mimics or NC oligonucleotide for 24 h. ^*∗*^ *P* < 0.05. (f) Western blot analysis for Mcl-1 protein level in HCC cells after transfection with miR-26b mimics or NC oligonucleotide for 24 h.

To investigate the biological role of miR-26b in HCC cells, we first measured the levels of Mcl-1 and mature miR-26b in four HCC cell lines (HepG2, Hep3B, PLC, and Huh7) and LO2 cells which are derived from human embryo liver and described as the normal hepatocytes, although they are immortalized [23]. The results showed that the HCC cell lines expressed lower level of miR-26b (Figure 1(a)) and higher Mcl-1 at both mRNA and protein levels (Figures 1(b) and 1(c)) compared with L02 cells, suggesting that there was an inverse relationship between the expressions of Mcl-1 and miR-26b, and the downregulation of miR-26b and upregulation of Mcl-1 may be involved in hepatocellular carcinogenesis. To check the influence of miR-26b on the expression of Mcl-1, we transfected the HCC cells with miR-26b mimics or NC oligonucleotide. As shown in Figure 1(d), the quantity of miR-26b in miR-26b mimics groups was upregulated significantly compared to NC oligo groups in all of the HCC cells. Then, to assess whether miR-26b had a functional role in downregulation of endogenous Mcl-1 expression, the Mcl-1 expression was determined using qPCR and western blot. As shown in Figures 1(e) and 1(f), miR-26b significantly repressed the expression of Mcl-1 at mRNA and protein levels in all of the four HCC cells.

### 3.2. Mcl-1 mRNA 3′-UTR Is the Direct Target of miR-26b

**Figure 2:**
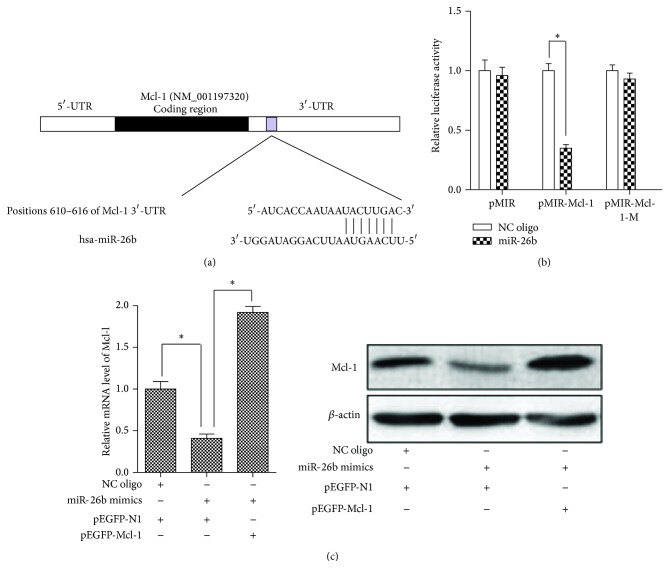
Mcl-1 mRNA 3′-UTR is the direct target of miR-26b. Three independent experiments were performed. (a) A predicted binding site of miR-26b in 3′-UTR of human Mcl-1 mRNA. (b) HepG2 cells were cultured in 48-well plates and were cotransfected with the reporter plasmids (pMIR-Mcl-1, pMIR-Mcl-1-M, or empty pMIR) and RNA oligos (miR-26b mimics or NC oligo) for 24 h. Then the luciferase activity was determined with Dual-Luciferase Reporter System. The expression of the reporter containing wild type 3′-UTR of Mcl-1 was suppressed by miR-26b, but not in the mutated construct or empty plasmid. ^*∗*^ *P* < 0.05. (c) qPCR and western blot analysis for Mcl-1 levels in HepG2 cells after transfection with RNA oligos (miR-26b mimics or NC oligo) and plasmid (pEGFP-N1 or pEGFP-Mcl-1) for 24 h. The suppression of Mcl-1 by miR-26b was abolished by transfection of the cells with pEGFP-Mcl-1 without 3′-UTR. ^*∗*^ *P* < 0.05.

All of the above results suggested that there was an inverse relationship between the expressions of Mcl-1 and miR-26b. So we tried to search for the target genes of miR-26b using TargetScan (http://www.targetscan.org/), and Mcl-1 was chosen finally for it contains putative miR-26b target sites in its 3′-UTR (UACUUGA, nt 610–616, Figure 2(a)). To directly test whether Mcl-1 was targeted by miR-26b, we cloned the 3′-UTR fragment of Mcl-1 into the pMIR reporter plasmid downstream of luciferase. Then, the luciferase reporter assays were performed in HepG2 cells with miR-26b mimics (or NC oligo) and reporter plasmids. As shown in Figure 2(b), miR-26b significantly reduced the luciferase activity of pMIR-Mcl-1 with the wild type 3′-UTR of Mcl-1. However, miR-26b did not affect the luciferase activity of pMIR-Mcl-1-M and empty pMIR. Moreover, transfection with pEGFP-Mcl-1 could totally overcome the suppression of Mcl-1 caused by miR-26b because the pEGFP-Mcl-1 contained no 3′-UTR (Figure 2(c)). Taken together, our data indicated that Mcl-1 3′-UTR is the direct target of miR-26b and suggested that miR-26b could suppress the expression of Mcl-1.

### 3.3. miR-26b Sensitized TRAIL-Induced Viability Inhibition and Apoptosis in HepG2 Cells

**Figure 3:**
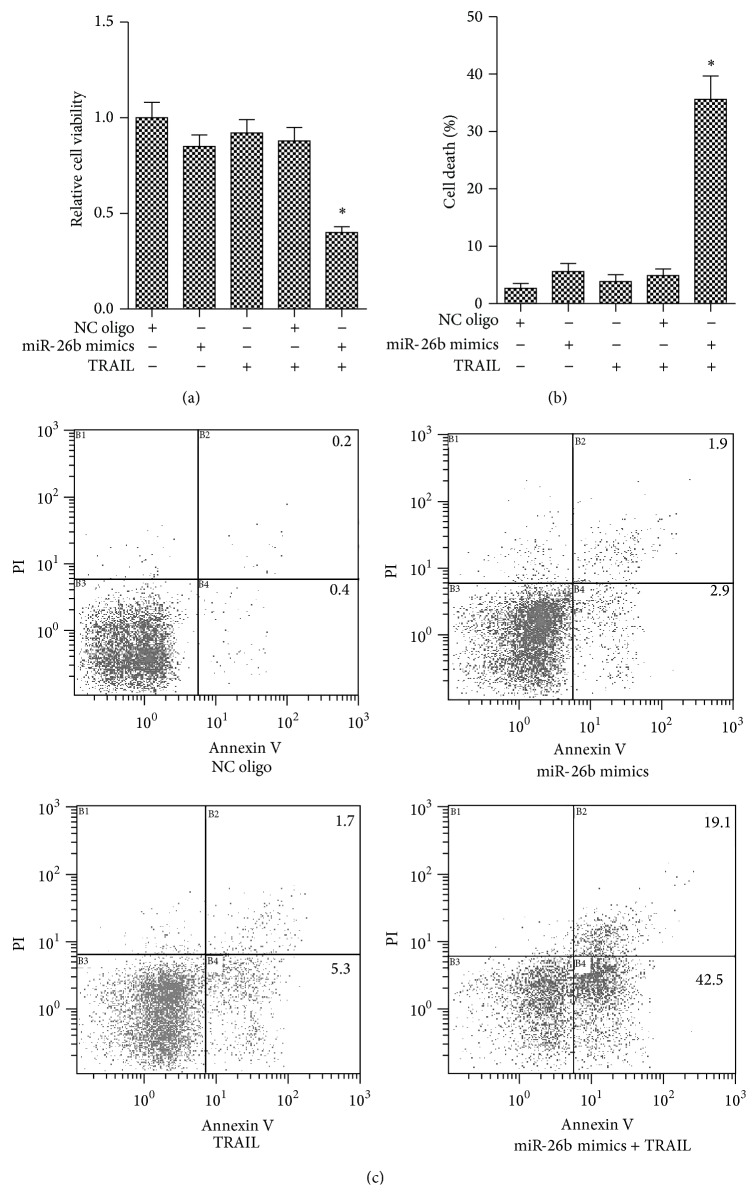
miR-26b sensitized TRAIL-induced cell viability inhibition and apoptosis in HepG2 cells. Three independent experiments were performed. (a) HepG2 cells were transfected with indicated RNA oligos with/without TRAIL. Then the MTT assay was performed for evaluating the cell viability. ^*∗*^ *P* < 0.05 versus NC oligo group. (b) HepG2 cells were transfected with indicated RNA oligos with/without TRAIL. Then the trypan blue exclusion assay was performed for evaluating the cell death. ^*∗*^ *P* < 0.05 versus NC oligo group. (c) HepG2 cells were transfected with indicated RNA oligos with/without TRAIL. Then the cell apoptosis was measured using Annexin V/PI staining on flow cytometry.

To study the role of miR-26b in apoptosis regulation in HCC cells, we treated with TRAIL which is an apoptotic stimulus after transfection with miR-26b in HepG2 cells. As shown in Figures 3(a) and 3(b), obvious cell viability inhibition and more cell death were observed in the combination group than in the control. However, in the groups treated with either miR-26b mimics or TRAIL alone, no significant cytotoxicity was observed. Then, the treated cells were collected and detected the apoptosis using Annexin V/PI staining on flow cytometry. As shown in Figure 3(c), HepG2 cells were resistant to TRAIL-induced apoptosis, as a low quantity of Annexin V positive cells was observed with flow cytometry. However, significant increase of apoptotic cells was observed in the sample treated with the combination of TRAIL and miR-26b mimics. These data suggest that overexpression of miR-26b would sensitize the cells to TRAIL cytotoxicity.

### 3.4. Exogenous Mcl-1 Abolished the Sensitization of miR-26b to TRAIL-Induced Cytotoxicity

**Figure 4:**
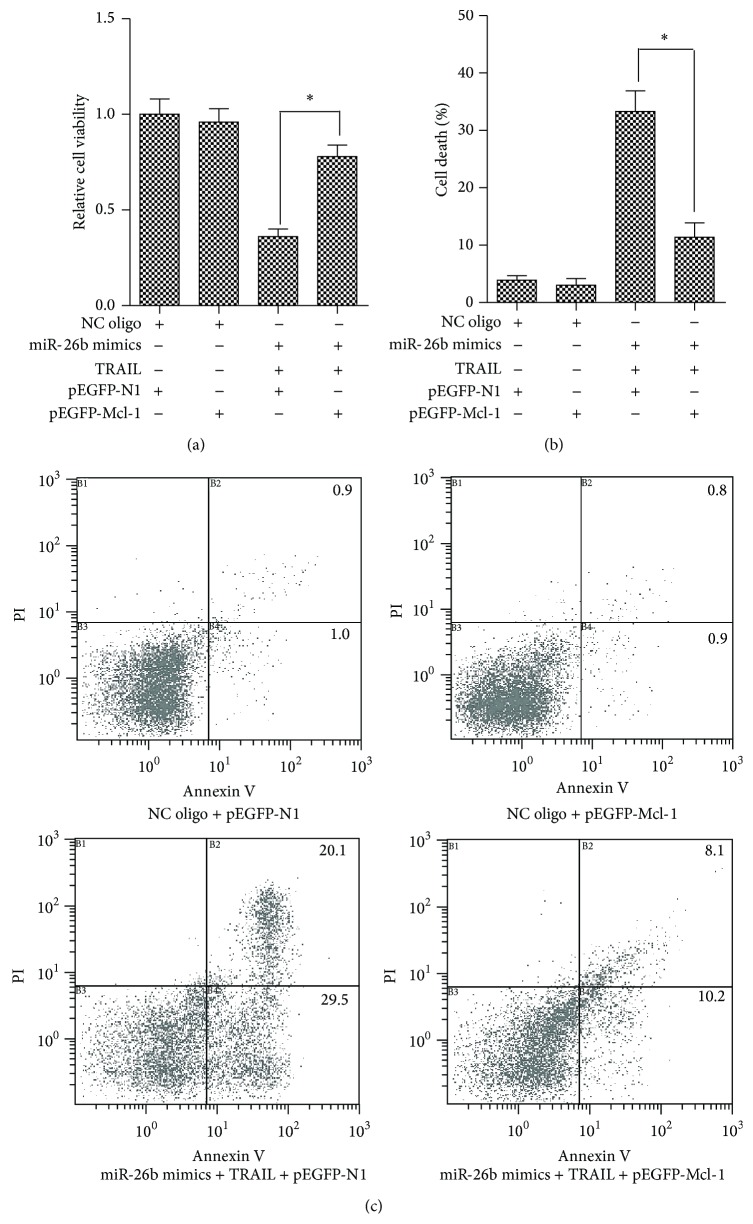
Mcl-1 abolished the sensitization of miR-26b to TRAIL-induced cytotoxicity. Three independent experiments were performed. (a) HepG2 cells were transfected with indicated RNA oligos with/without TRAIL. Then the MTT assay was performed for evaluating the cell viability. ^*∗*^ *P* < 0.05 versus NC oligo group. (b) HepG2 cells were transfected with indicated RNA oligos with/without TRAIL. Then the trypan blue exclusion assay was performed for evaluating the cell death. ^*∗*^ *P* < 0.05 versus NC oligo group. (c) HepG2 cells were transfected with indicated RNA oligos with/without TRAIL. Then the cell apoptosis was measured using Annexin V/PI staining on flow cytometry.

Mcl-1 is an antiapoptotic protein in the Bcl-2 family members, and downregulation of Mcl-1 induced cell growth inhibition in HCC cells [24]. Therefore, we speculated that the mechanism of miR-26b sensitized TRAIL-induced cytotoxicity was due to the downregulation of Mcl-1 caused by miR-26b. So we cotransfected with miR-26b and pEGFP-Mcl-1 before being treated with TRAIL in HepG2 cells. As shown in Figure 4, transfection of pEGFP-Mcl-1 significantly inhibited the cytotoxicity of miR-26b combining with TRAIL. Taken together, the data support a role for miR-26b-mediated Mcl-1 regulation that modulates cellular sensitivity to TRAIL.

## 4. Discussion

Previous researches have demonstrated that Mcl-1 is a key antiapoptotic protein in HCC cells. Sieghart et al. [9] showed that the tumor specimens overexpressed Mcl-1 in HCC patients; however, in paired samples of nontumor liver tissue adjacent to HCC, no positive staining was observed even if they were directly neighbouring tumor tissue, suggesting that the overexpression of Mcl-1 is tumor specific. Moreover, since liver tissue specific deletion of Mcl-1 does not induce apoptosis of normal hepatocytes, targeting Mcl-1 for HCC therapy might not harm healthy liver tissue [25, 26]. Mcl-1 also plays an important role in other cancer cells. Thallinger et al. [27] reported that Mcl-1 antisense oligonucleotides treatment of SCID mice with melanoma subcutaneous tumors resulted in cancer sensitization to the chemotherapeutic drugs, accompanied by increased levels of apoptosis in tumor cells. Pancreatic adenocarcinoma is another cancer that overexpresses Mcl-1. Knockdown of Mcl-1 led to the cell death in a pancreatic cancer cell line, and loss of Mcl-1 sensitized the cancer cells to Gemcitabine [28]. These studies suggested that Mcl-1 may become a prospective target for cancer therapy.

MicroRNAs are endogenous and effective small molecules to influence multiple physiological processes via regulating relative gene expression. Recent studies show that the expression changes of miRNAs always lead to tumorigenesis [29]. Increasing evidence suggests that microRNAs play a role in carcinogenesis by altering transcription of oncogenes and tumor suppressor genes to promote the proliferation and metastasis as well as antiapoptosis for cancer cells [30–33]. Therefore, subsets of miRNAs have been identified as potential diagnostic and prognostic markers in malignant tumours [34]. miR-26b has been reported to have extensive biological effects in cells [35], and recent studies prove that miR-26b was always downregulated in multiple cancers. Li et al. [36] indicated that miR-26b plays a role as tumour suppressor gene in breast cancer by targeting the CDK8. Similarly, the miR-26b level was downregulated in HCC, which inhibits the epithelial-mesenchymal transition and cell proliferation [37].

In this study, we demonstrate that miR-26b plays a role as a tumor suppressor in HCC cells. HCC cell lines expressed higher Mcl-1 and lower level of miR-26b compared with L02 cells, indicating that there was an inverse relationship between the expressions of Mcl-1 and miR-26b. Our data proved that miR-26b was a novel regulator of Mcl-1, through binding to 3′-UTR of Mcl-1 mRNA. Downregulation of Mcl-1 mediated by miR-26b significantly sensitized TRAIL-induced viability inhibition and apoptosis in HepG2 cells and exogenous Mcl-1 abolished the sensitization of miR-26b to TRAIL-induced cytotoxicity.

Our study provides the first evidence that miR-26b was a novel regulator of Mcl-1, and Mcl-1 plays a key role in the sensitization effect of miR-26b on TRAIL-induced apoptosis in HCC in vitro. These results also suggested that miR-26b/Mcl-1 pathway might act as a sensitizer for chemotherapy and may be a novel target for the treatment of HCC.
